# Renewable Green Platform Chemicals for Polymers

**DOI:** 10.3390/molecules22030376

**Published:** 2017-02-28

**Authors:** Parijat Ray, Craig Smith, George P. Simon, Kei Saito

**Affiliations:** 1 Department of Materials Science and Engineering, Monash University, Clayton, VIC 3800, Australia; parijat.ray@monash.edu; 2 PPG Australia, McNaughton Rd, Clayton, VIC 3168, Australia; craig.smith@ppg.com; 3 School of Chemistry, Monash University, VIC 3800, Australia

This Special Issue covered topics in the field of Green Chemistry. One of the 12 principles in Green Chemistry is “Safer Solvents and Auxiliaries” which is reasonably covered in this Special Issue and examples are shown using ionic liquids [1], water [2], and a solvent-free system [3]. Another principle, “Catalysis”, is also covered, showing that novel heterogeneous catalysts can be used in various reactions [4,5,6]. Non-covalent derivatives that can be applied to proceed “Less Hazardous Chemical Syntheses” are presented by one of the founders of Green Chemistry (J.C. Warner) in this Special Issue [7].

The principle “Use of Renewable Feedstocks” is not well covered in this Special Issue but it is important, especially for the green polymer syntheses. In the modern world, the polymer plays a ubiquitous role. Our day-to-day life is completely pervaded by polymers, most of which are derived from petrochemical sources. The continuously increasing demand for fossil fuels and associated environmental concerns has led research to identify alternative materials which are renewable, green and sustainable in terms of resourcing, production and end-use-applications. Biomass, from both land and sea, seems to be the most promising alternative to petrochemical-based resources in terms of renewability, production and impact on the environment [8].

To date, different types of renewable resources have been reported which can be used to make polymers, and some of their end products are already commercialized using natural polymers such as cellulose and its derivatives, silk, cotton, chitin and chitosan [9]. There are some other examples which also seem promising to assist in dealing with the challenges of renewable polymer productions. Itaconic acid (IA) is a naturally occurring organic compound which is non-toxic and is a promising renewable source for the synthesis of bio-based polymers [10]. By using the functional carboxylic acid groups, it can be modified or copolymerized into different polymers such as epoxy, amide, polyesters etc. Itaconic acid also has a double bond which can undergo free radical polymerization to produce both homo- and co-polymers with other monomers. Previously, itaconic acid was obtained by the distillation of citric acid but currently it can be produced industrially by the fermentation of carbohydrates, such as glucose, using *Aspergillus terreus* [11]. It can also be used as a platform chemical to produce chemicals such as itaconic diamide, itaconic anhydride, 2-methyl-1,4-butanediol, 2-methyl-1,4-butanediamine from which different polymers can be obtained [12]. Another important bio-based platform chemical is 5-hydroxymethyl furfural (HMF) (derived from hexoses, mainly fructose). HMF and its derivatives such as 2,5-furandicarboxylic acid (FDCA) have been listed as a “Top 10+4” bio-based chemical evaluated by U.S Department of Energy (DOE) [13]. HMF is considered as a strong platform chemical as it can be converted into different sustainable chemicals such as alkoxymethylfurfurals, 2,5-furandicarboxylic acid, 5-hydroxymethylfuroic acid (furanic derivatives) etc., and levulinic acid, adipic acid, 1,6-hexanediol, caprolactum and caprolactone (non-furanic derivatives) which can be used to make different polymers [14]. FDCA is found to be good replacement of terephthalic acid and is already being used commercially along with ethylene glycol to make 100% bio-based polyester [15]. The chemical structure of glycerol makes it a very interesting platform chemical for conversion into other chemicals and it can also be used directly in polycondensation reactions to make polyesters [16]. Depending on the modification, it can be converted into huge numbers of chemicals, such as tartronic acid, hydroxyethanoic acid and hydroxypyruvic acid (by oxidation reaction) for polyester synthesis; ethylene glycol, 1,2-propandiol and 1,2-propanediol (by hydrogenolysis reaction) for polyester and polyurethane synthesis; glycerol dimethacrylate (by transesterification reaction) for polyacrylate synthesis [17]. 3-Hydroxypropanoic acid (HPA) is also an interesting platform chemical which can be derived from glucose by enzymatic catalysis or fermentation. The bifunctionality of this chemical allows it to be be used to synthesize different platform chemicals for polymers by simple chemical transformation. It can be converted into 1,3-propanediol, malonic acid and 3-hydroxypropylamide for polyesters, and glycerol dimethylacrylate and glycidol for acrylates and epoxies [18]. HPA can be coupled with other acids to produce polyesters such as polytrimethylene terephthalate which has huge applications in fiber industries [19]. HPA can itself undergo a polycondensation reaction to form poly(3-hydroxypropanoic acid) which has a melting temperature of 77 °C and glass transition temperature of −22 °C. Levulinic acid (4-oxopentanoic acid) can be produced from lignocellulose waste at a low cost and is considered to be an important platform chemical because of its reactive nature [20]. Levulinic acid esters are used in food industries, as solvents and as plasticizers. It can be converted into 4-hydroxy pentanoic acid through hydrogenation reaction and 1,4-pentanediol which can be used to make polyesters [21]. α-Methylene-γ-valerolactone, which can be derived from levulinic acid, can undergo free radical polymerization and is thought to be an alternative to methylmethacrylate (MMA) [22].

There are other different platform chemicals such as lactides, and 1,4-diacids such as succinic and fumaric acids, glutamic and glucaric acids and sorbitols which can be converted into their corresponding counterparts with appropriate functional group modification, then transformed into the respective polymers [23]. Still, challenges remain in terms of the production of platform chemicals for polymers at large scale with low cost and sustainable conditions.
